# Thiacetazone, an Antitubercular Drug that Inhibits Cyclopropanation of Cell Wall Mycolic Acids in Mycobacteria

**DOI:** 10.1371/journal.pone.0001343

**Published:** 2007-12-19

**Authors:** Anuradha Alahari, Xavier Trivelli, Yann Guérardel, Lynn G. Dover, Gurdyal S. Besra, James C. Sacchettini, Robert C. Reynolds, Geoffrey D. Coxon, Laurent Kremer

**Affiliations:** 1 Laboratoire de Dynamique des Interactions Membranaires Normales et Pathologiques, Université de Montpellier II et I, Centre National de la Recherche Scientifique (CNRS), UMR 5235, Montpellier, France; 2 INSERM, Dynamique des Interactions Membranaires Normales et Pathologiques (DIMNP), Montpellier, France; 3 Unité de Glycobiologie Structurale et Fonctionnelle, CNRS UMR 8576, Université des Sciences et Technologies de Lille, Villeneuve d'Ascq, France; 4 Biomolecular and Biomedical Research Centre, School of Applied Science, Northumbria University, Newcastle upon Tyne, United Kingdom; 5 School of Biosciences, University of Birmingham, Edgbaston, Birmingham, United Kingdom; 6 Department of Biochemistry and Biophysics, Texas A&M University, College Station, Texas, United States of America; 7 Drug Discovery Division, Southern Research Institute, Birmingham, Alabama, United States of America; 8 Division of Pharmaceutical Sciences, Strathclyde Institute of Pharmacy and Biomedical Sciences, University of Strathclyde, Glasgow, United Kingdom; Cairo University, Egypt

## Abstract

**Background:**

Mycolic acids are a complex mixture of branched, long-chain fatty acids, representing key components of the highly hydrophobic mycobacterial cell wall. Pathogenic mycobacteria carry mycolic acid sub-types that contain cyclopropane rings. Double bonds at specific sites on mycolic acid precursors are modified by the action of cyclopropane mycolic acid synthases (CMASs). The latter belong to a family of *S*-adenosyl-methionine-dependent methyl transferases, of which several have been well studied in *Mycobacterium tuberculosis*, namely, MmaA1 through A4, PcaA and CmaA2. Cyclopropanated mycolic acids are key factors participating in cell envelope permeability, host immunomodulation and persistence of *M. tuberculosis*. While several antitubercular agents inhibit mycolic acid synthesis, to date, the CMASs have not been shown to be drug targets.

**Methodology/Principle Findings:**

We have employed various complementary approaches to show that the antitubercular drug, thiacetazone (TAC), and its chemical analogues, inhibit mycolic acid cyclopropanation. Dramatic changes in the content and ratio of mycolic acids in the vaccine strain *Mycobacterium bovis* BCG, as well as in the related pathogenic species *Mycobacterium marinum* were observed after treatment with the drugs. Combination of thin layer chromatography, mass spectrometry and Nuclear Magnetic Resonance (NMR) analyses of mycolic acids purified from drug-treated mycobacteria showed a significant loss of cyclopropanation in both the α- and oxygenated mycolate sub-types. Additionally, High-Resolution Magic Angle Spinning (HR-MAS) NMR analyses on whole cells was used to detect cell wall-associated mycolates and to quantify the cyclopropanation status of the cell envelope. Further, overexpression of *cmaA2*, *mmaA2* or *pcaA* in mycobacteria partially reversed the effects of TAC and its analogue on mycolic acid cyclopropanation, suggesting that the drugs act directly on CMASs.

**Conclusions/Significance:**

This is a first report on the mechanism of action of TAC, demonstrating the CMASs as its cellular targets in mycobacteria. The implications of this study may be important for the design of alternative strategies for tuberculosis treatment.

## Introduction

A serious concern in antitubercular therapy is the emergence of multi-drug resistant (MDR) strains, and more recently, extensively drug-resistant (XDR) strains of *Mycobacterium tuberculosis* (*M. tb*) [1]. Strains of *M. tb* resistant to isoniazid and rifampicin, important components in the first-line of drug treatment, are categorized as MDR, while XDR strains of *M. tb* are defined as those that are also resistant to at least three of the six classes of second-line drugs (aminoglycosides, polypeptides, fluoroquinolones, thioamides, cycloserine and *p*-aminosalicylic acid), which seriously limits treatment options [2]. According to a recent WHO report, 10% of MDR cases were XDR, across all geographical regions surveyed, thus posing the threat of an untreatable global epidemic [3]. Therefore, the need for rapid and continued progress in understanding the mechanism of action of the current antitubercular agents and the discovery of new cellular drug targets remain ever present.

Thiacetazone (TAC) is an inexpensive, antitubercular, bacteriostatic drug that has been widely used in combination with isoniazid in Africa and South America [4]. Chemical analogues of TAC, SRI-224 and SRI-286, have been synthesized and tested against *Mycobacterium avium* and found to be more effective than TAC *in vitro* and in mice [5]. We and others have recently shown that TAC is a prodrug that is activated by the mycobacterial monooxygenase EthA, which is also the activator of two other anti-tuberculosis drugs, ethionamide (ETH) and isoxyl (ISO) [6], [7], [8]. However, the mechanism of action of TAC remains an enigma. Our first observation on effects of TAC on *M. bovis* BCG was that it affects mycolic acid synthesis [6].

**Figure 1:**
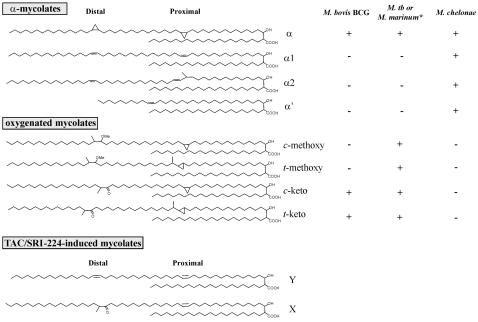
Structures and occurrence of mycolic acid sub-types in mycobacterial species presented in this study. * proximal position of oxygenated mycolates is unsaturated [37].

Mycolic acids are very long chain, α-alkyl, β-hydroxy fatty acids (Figure 1), covalently linked to arabinogalactan or trehalose in the complex cell walls of bacteria of the *Corynebacterium-Mycobacterium-Nocardia* group [9], [10]. These lipids give rise to important characteristics including resistance to chemical injury and dehydration, low permeability to antibiotics, virulence [11], [12], [13], acid-fast staining [14] and the ability to persist within the host [12], [14], [15], [16], [17]. Mycolic acids are also the targets of front-line antitubercular drugs, such as isoniazid (INH) and ETH [18], [19], [20]. Mycolates from mycobacteria possess the longest carbon chains consisting of the C_56 _meromycolate and the C_26 _α-branch. Their synthesis is brought about by the co-ordinate activities of several enzymes, involving numerous biochemical steps. During synthesis, the meromycolate intermediate, which is interrupted by double bonds at specific sites, may be modified by the action of different *c*yclopropane *m*ycolic *a*cid *s*ynthases (CMASs) that convert double bonds to cyclopropane rings. These modifications occur at the sites of the two characteristic double bonds incorporated in the chain, the proximal (closer to the β-hydroxy) or the distal double bond [9], [10]. Enzymes of the CMAS family are *S*-adenosylmethionine-dependent (SAM) methyltransferases, that share a high degree of sequence and structural homology [21], [22]. In *M. tb*, MmaA2 [23] and PcaA [12] modify these double bonds to *cis*-cyclopropanes to produce α-mycolates. Synthesis of both keto- and methoxy-mycolates involves oxidation, methylation and *cis*- or *trans*-cyclopropanation by the coordinate activities of the MmaA1 through MmaA4 and CmaA2 [9]. Differential action of the various CMASs leads to generation of a repertoire of mycolic acid sub-types [22], [24], [25], [26], [27].

Molar ratios of the mycolic acid sub-types present in the mycobacterial cell wall is species-dependent and has a profound effect on the fluidity and permeability of the cell wall as well as the immunological response in the host [11], [12], [28], [29]. Mycolic acids are either covalently attached to arabinogalactan or non-covalently associated with the wall as complex glycolipids like trehalose dimycolate (TDM), also called cord factor. While the latter is an important immunomodulator in pathogenic mycobacteria, it has been recently shown that its proinflammatory capacity is dependent on the nature of the constituent mycolic acids. For example, TDM extracted from the cell envelope of a *pcaA* knock-out mutant, was deficient in *cis*-cyclopropanated α-mycolic acids and was shown to be hypoinflammatory [12]. The *pcaA* mutant was also compromised in long-term survival in host mice. In sharp contrast, a null mutant in the *cmaA2* gene of *M. tb* that lacked *trans*-cyclopropanated mycolates, was hypervirulent and the proinflammatory response was transferable through its TDM [29]. A *mmaA4* knockout mutant that lacks keto- and methoxy-mycolates, showed reduced cell wall permeability and was also attenuated in mice [11]. These reports illustrate the fact that enzymes of the CMAS family are particularly relevant to virulence and persistence of *M. tb*.

In this study, we present a chemical library of twenty-three analogues of TAC. Also, for the first time, a molecular target of TAC and its chemical analogue SRI-224 is revealed. We have analyzed the changes in mycolic acid profile in TAC-treated cells of different mycobacterial species. An accumulation of uncyclopropanated mycolates was observed. This was further confirmed by whole cell analyses, using a recently introduced application of HR-MAS NMR. Further, strains independently overexpressing *cmaA2*, *mmaA2* or *pcaA* were constructed and found to be significantly less affected by TAC or SRI-224 in their mycolate profile. Thus, our results indicate that these drugs alter mycolic acid biosynthesis by inhibiting cyclopropanation of the meromycolate chain. The lack of cyclopropanation of mycolates in drug-treated mycobacteria is likely one of the reasons for the observed effectiveness of the TAC *in vivo*. Nevertheless, presence of other cellular targets of the drug, possibly, other SAM-dependent enzymes, is not ruled out.

## Methods

### Mycobacterial strains and growth conditions

*M. bovis* BCG strain Pasteur, *M. chelonae* (ATCC 19536), and *M. marinum* M strain were all grown either on Middlebrook 7H10 agar supplemented with oleic acid-dextrose-catalase (OADC) enrichment or in Sauton's broth medium at 37°C or at 30°C for *M. marinum*. Mycobacteria were transformed by electroporation and recombinant clones were selected on 7H11 supplemented with OADC and 25 µg/ml kanamycin.

### Drug susceptibility testing of *M. tuberculosis*

**Figure 2:**
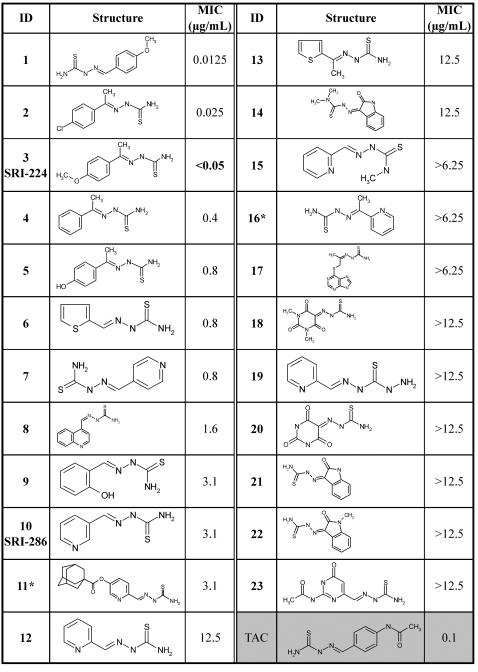
Structures of chemical analogues of thiacetazone and their corresponding minimum inhibitory concentrations (MICs) in *M. tb* H37Rv. MICs were determined by BACTEC 460 radiometric assay. *Data from MABA assay.

TAC was purchased from Sigma (Saint Louis, MO), while SRI-224 and other chemical analogues of TAC were synthesized at the Southern Research Institute (Birmingham, AL). The chemical synthesis of the analogues will be documented separately. A primary screen was conducted by the BACTEC 460 radiometric assay at an initial screening concentration of 12.5 µg/ml against *M. tb* H_37_Rv (ATCC 27294) in BACTEC 12B medium as reported [30]. Two samples (entries **11** and **16**, Figure 2) were screened against *M. tb* H_37_Rv in BACTEC 12B medium using the Microplate Alamar Blue Assay (MABA) [30]. Drug stocks were prepared in DMSO for dilution, and the stocks were filter sterilized and stored at –70°C until usage. Compounds demonstrating at least 90% inhibition in the primary screen were tested again at lower concentrations in the BACTEC 460 system against *M. tb* H_37_Rv to determine the minimum inhibitory concentration (MIC). The MIC is defined as the lowest concentration effecting a reduction in fluorescence of 99% relative to controls. For entry **11** in Figure 2, MIC_90_ was derived using the MABA assay. It has been reported that the MABA MIC_90_ correlates well with a MIC_99_ for the BACTEC 460 assay [30].

### Plasmids and genetic manipulations

All cloning steps were performed in *Escherichia coli* TOP-10 (Invitrogen). *M. tb* H37Rv genomic DNA was used as a template to amplify the coding regions of *cmaA2*, *mmaA2* and *pcaA* using either Vent DNA polymerase (New England Biolabs) or the Phusion High-fidelity enzyme (Finnzymes). The following pairs of primers were used: *cmaA2*-fwd 5′-TGA CGT CAC AGG GCG ACA CGA CAA GCG G-3′, *cmaA2*-rev 5′-GGA ATT CTT ATT TGA CCA GAG TGA ACT GGC-3′, *mma2*-fwd 5′-TGG TCA ACG ACC TAA CGC CGC AC-3′, *mmaA2*-rev 5′-GCG CAA GCT TCT ACT TGC CAG CGT GAA CTG G-3′, *pcaA*-fwd 5′-TGT CCG TGC AGC TCA CGC CGC-3′, *pcaA*-rev 5′-GGA ATT CTT ACT TTT CCA GTG TGA ACT GGT CG-3′. The underlined sequences indicate the *Eco*RI or *Hin*dIII restriction sites used in cloning the PCR products into plasmid pMV261 double-digested with the same enzyme and *Msc*I. This allowed the genes to be expressed constitutively from the *hsp60* promoter of the vector [31]. All restriction enzymes were purchased from New England Biolabs. All plasmid constructs were validated by DNA sequencing (MWG Biotech).

### Determination of the *in vivo* effects of TAC and analogues on mycolic acid synthesis

The different mycobacterial strains were grown in Sauton's medium and treated with varying concentrations of TAC or its analogues as discussed in the text. *In vivo* radiolabeling of lipids of was performed with [1, 2-^14^C]-acetate (1 µCi/ml, 56 mCi/mmol, Amersham Biosciences) for 8 h. Cells were harvested, subjected to alkaline hydrolysis in tributyl-ammonium hydroxide as described previously [6], [33]. Methylesterification, extraction and resolution of mycolic acids by either normal phase or argentation or 2-D thin layer chromatography (TLC) was carried out as described previously [6], [33]. Briefly, equal radioactive amounts of each sample were applied to a silica-coated aluminium TLC plate, which was developed in different mixtures of solvents depending on the nature of the TLC. TAC or SRI-224 solutions were added to the growth medium at desired concentrations for a total period of 24 h, prior to and including the 8 h of *in vivo* labeling period. In separate experiments, L-[*methyl*-^14^C]-methionine (1 µCi/ml, 57 mCi/mmol, Amersham Biosciences) was added to the cultures. For the identification of mycolic acids covalently attached to the cell wall, cells were first delipidated with chloroform/methanol (2∶1) to remove non-covalently attached lipids as reported earlier [33], followed by methylesterification and extraction in diethyl ether. Mycolates were finally dissolved in dichloromethane prior to TLC analysis.

### Large-scale purification of the lipids X and Y

For structural analysis, mycobacterial lipids X and Y were purified from 4L of *M. bovis* BCG shake-flask cultures, treated with 5 µg/ml SRI-224 for 24 h [6]. Extraction of the mycolic acid methyl esters (MAMEs) was carried out as described above. These were applied to a column of silica coated with 50% solution of silver nitrate and activated at 95°C, overnight. The MAMEs were eluted from the column with a gradient of increasing concentrations of diethyl ether, from 2% to 90%, in petroleum ether. The various fractions were analyzed by argentation TLC. Lipids X and Y were identified by their behavior on argentation TLC and verified by comparison with radiolabeled mycolates extracted from drug-treated cells. Fractions containing X and Y were applied to a preparative TLC plate developed in petroleum ether:acetone (19∶1, v/v) and the products were scraped off the plate and resuspended in diethyl ether. The solvent was then dried and the purified lipids resuspended in dichloromethane. Purity was verified by argentation TLC.

### Mass Spectrometry

Mass spectrometric analyses of the methyl esters of lipids X or Y from M. bovis BCG, purified as above, were done on a Voyager Elite reflectron MALDI-TOF mass spectrometer (PerSeptive Biosystems, Framingham, MA, USA), equipped with a 337 nm UV laser. Samples were solubilized in 1 µl chloroform/methanol (2∶1) and mixed on target with 1 µl of 2, 5-dihydroxybenzoic acid matrix solution (10 mg/ml dissolved in chloroform/methanol 2∶1).

### NMR analyses

Lipids X or Y from *M. bovis* BCG, purified as described above, were dissolved into deuterated chloroform containing 0.01% of TMS and transferred into Shigemi tubes matched for D_2_O. Then 0.1 ml of deuterium oxide was added to avoid solvent evaporation during long acquisition [34]. 1D proton NMR spectra were recorded at 299K on a 800 MHz Avance II and 400 MHz Avance Bruker spectrometers equipped with a ^1^H/^13^C/^15^N/^2^H and a broad-band probes, respectively.

### Whole cell analyses by HR-MAS NMR

*M. bovis* BCG cultures grown in Sauton's medium were treated with 1 µg/ml of either TAC or SRI-224 for 5 days. Cells were harvested by centrifugation, heat-inactivated at 80°C for 20 min and stored at 4°C till further use. For HR-MAS NMR analysis, cell pellets were washed several times with deuterium oxide in order to remove protonated water. Four mm Zr rotors (CortecNet, France) were filled with 50 µl of cell pellets, centrifuged at low speed and stirred with 5 ml of chloroform. Experiments were recorded on a 800 MHz AvanceII Bruker spectrometer at 293K with a ^1^H/^13^C/^15^N/^2^H probe spinning at 8 kHz. Uni-dimensional selective COrrelation SpectroscopY (COSY) spectrum was recorded with 1024 scans, a 5 ms Gaussian selective excitation on the Ha resonance at −0.33 ppm and a 17.5 ms evolution delay.

## Results

### Structure of TAC-related analogues and their activity against *M. tuberculosis*

TAC has been widely used as a front-line therapeutic in poor countries, since it is inexpensive [35]. This drug may prove useful to treat patients infected with MDR strains, for whom the choice of effective drugs is limited. TAC, a thiocarbamide-containing drug, was chosen as a target pharmacophore for the preparation of a second generation analogues (chemical synthesis will be described elsewhere). We present here a library of 23 chemical analogues of TAC, including SRI-286 and SRI-224 [5], which have previously been shown to be active against *M. avium*. We have evaluated the potential of these drugs in inhibiting growth of *M. tb* H37Rv. Drug susceptibility of *M. tb* by determination of the minimal inhibitory concentration (MIC) of TAC and its analogues was carried out using the BACTEC 460 radiometric method. As shown in Figure 2, several analogues exhibited potent antitubercular activity. Although this set represented modest diversity, a few potential trends are notable. For example, three compounds (**1**, **2** and SRI-224) were found to be more active than the parent molecule, suggesting that newer, more effective analogues might be developed for clinical use. Entries **1**–**7** in Figure 2 show significant activity (<1.0 µg/ml). These are all simple arylmethyl thiosemicarbazones where the aryl group is a phenyl, thiophene, or pyridyl aromatic. The three most active compounds are 4-substituted phenyl analogues as is TAC. The 4-pyridyl analogue **9** is the most active of the pyridyl analogues (2-pyridyl, **15** or 3-pyridyl, **10**), and this analogue is, in many respects, similar to a 4-substituted phenyl analogue. It is notable that a methyl substitution **3** can retain significant activity relative to the des-methyl analogue **1**, although this trend is not without exception (**7** versus **16**). For the most part, larger aryl groups attached to the thiosemicarbazone by different distances/linkers results in compounds with reduced to little activity. However, larger numbers of analogues will be required to develop a robust structure-activity relationship for the thiosemicarbazone class of antitubercular agents.

### Inhibition of mycolic acid synthesis by TAC

We have recently reported that, following activation by the monooxygenase EthA, TAC strongly alters the mycolic acid profile of *M. bovis* BCG-treated cells [6]. Interestingly, this altered profile was radically different from that after exposure of *M. bovis* BCG to ISO, INH or ETH, all of which are antitubercular drugs known to inhibit mycolic acid biosynthesis [6]. This study was aimed at extending our previous observations to other mycobacterial species and includes chemical analogues of TAC in order to identify the specific enzyme(s) of the mycolic acid pathway that are inhibited by these thiocarbamide-containing drugs.

**Figure 3:**
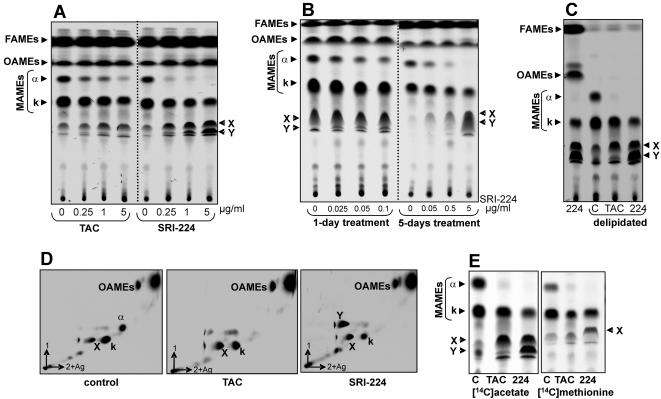
Inhibition of mycolic acid biosynthesis in *M. bovis* BCG by treatment with TAC or its analogue SRI-224. Exponentially-growing cultures were treated with the drugs for 18 h and labeled by adding ^14^C-acetate for another 8 h. Fatty acid methyl esters (FAMEs) and mycolic acid methyl esters (MAMEs) were then extracted and separated by TLC on 10% silver nitrate-impregnated plates prior to exposure to a film overnight. All extracts were loaded equally for 100,000 cpm on silica plates impregnated with 10% silver nitrate. The autoradiographs show FAMEs, MAMES, oleic acid methyl esters (OAMEs), α- and keto-mycolates (k) and the lipids X and Y as indicated by arrowheads. (A) 1D TLC analysis using petroleum ether and diethyl ether (17∶3, v/v) as solvents. Drug concentrations employed are indicated in µg/ml. (B) 1D TLC profile of MAMEs extracted from cells treated with low concentrations of SRI-224 for either 1 day or over a period of 5 days, as indicated. (C) Extracts prepared after delipidation of the cells to remove the free and loosely bound lipids, while retaining the covalently bound mycolates. Extract from cells treated with SRI-224 but not subjected to delipidation is included to identify the lipids X and Y by comparison with extracts from delipidated cells that were either untreated (c) or treated with 5 µg/ml of the indicated drug for 24 h. (D) 2D TLC analysis on silica plates impregnated with 10% silver nitrate. Extracts were separated in the first direction by using two developments with hexane/ethyl acetate (19∶1, v/v) and in the second direction by using a triple development with petroleum ether/diethylether (17∶3, v/v). (E) Extracts from cells radiolabeled with [*methyl*-^14^C]-methionine are compared with those from cells radiolabeled with ^14^C-acetate.

The effect of treatment of TAC or its chemical analogue, SRI-224 (**3** in Figure 2) on cell wall mycolates of *M. bovis* BCG strain Pasteur was examined. Cells in the exponential phase of growth were treated with increasing drug concentrations and labeled with ^14^C-acetate. Fatty acid methyl esters (FAMEs) and mycolic acid methyl esters (MAMEs) were extracted, resolved by TLC and visualized by autoradiography as described earlier [6]. Resolution of the extracts by conventional TLC did not reveal major changes in the mycolic acid profile after drug treatment (data not shown). However, differences were apparent when the extracts were resolved on TLC plates impregnated with silver nitrate. This method of argentation TLC retards the migration of unsaturated FAMEs and MAMEs [33]. Treatment of the cells with TAC or SRI-224 led to a significant reduction in the synthesis of the α-mycolates and to a lesser extent in that of the keto-mycolates, while synthesis of oleic acid remained unaffected (Figure 3A). At TAC concentrations between 1–5 µg/ml, accumulation of a product, designated lipid X, was detectable. Treatment with SRI-224 produced an additional radiolabeled product, designated lipid Y. Among the analogues, SRI-224 appeared to be the most effective in terms of inhibition of mycolate synthesis. Although exposure to SRI-286 (**10** in Figure 2) was also accompanied by a clear reduction in the synthesis of α- and keto-mycolates, this effect was less dramatic than after exposure to TAC or in SRI-224 (data not shown), consistent with the higher MIC value of SRI-286 against *M. bovis* BCG strain Pasteur (MIC_99_ = 10–25 µg/ml for SRI-286, 0.25 µg/ml for SRI-224, 0.5 µg/ml for TAC, [6]). Inhibitory effects on mycolic acid syntheses were observed even at SRI-224 concentrations lower than the MIC value (Figure 3B). These data suggest that the level of mycolate inhibition, as observed on argentation TLC, is directly correlated to the susceptibility of *M. bovis* BCG towards the TAC analogues. Moreover, treatment with the drug at low concentrations for a longer period of time, such as 5 days, accentuated these effects (Figure 3B).

Mycolates can either be covalently attached to arabinogalactan or present as free lipids when associated with sugars such as trehalose [9], [10]. We investigated whether the accumulating lipids, X and Y are attached to arabinogalactan or are present as extractable lipids within the cell wall. Drug-treated or untreated control cells were delipidated to remove lipid components that are non-covalently attached to the cell wall and retain only those that are covalently bound to arabinogalactan [33]. The lipids X and Y were still present in the delipidated cells as seen in Figure 3C, suggesting that they are covalently bound to the cell wall. Further, when resolved by two-dimensional argentation TLC (Figure 3D), the accumulating product X was found to migrate in the first dimension along with the oxygenated keto-mycolates, and Y with the α-mycolates.

*In vivo* radiolabeling with ^14^C-acetate labels all sub-types of mycolic acids. In contrast, labeling with [*methyl*-^14^C]-methionine would reveal only those mycolates that involve *S*-adenosylmethionine (SAM)-dependent methylation during their synthesis. Thus, both, the α- and keto-mycolates are labeled due to the presence of cyclopropane rings and additional methyl groups on the keto-mycolates (Figure 1). Synthesis of the mycolates in the presence of TAC or SRI-224, as visualized by labeling with [*methyl*-^14^C]-methionine, indicated that the drug-induced product X is labeled, but Y is not (Figure 3E). We therefore hypothesize that product Y is a di-unsaturated lipid, devoid of cyclopropanes, while X likely carries a methyl group. Taken together, these data suggest that X and Y are unsaturated counterparts of the keto- and α-mycolates, respectively.

### Structural determination of the drug-induced mycolate precursors, X and Y

**Figure 4:**
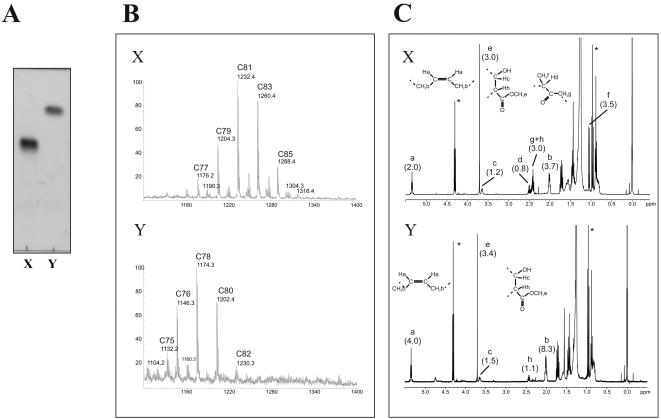
Structural determination of lipids X and Y. Lipids X and Y were purified from cell wall extract of *M. bovis* BCG Pasteur culture, following treatment with SRI-224 (5 µg/ml) for 24 h. (A) Conventional TLC showing purity of the samples containing lipids X and Y, that were used for structural analyses, as seen by staining with phosphomolybdic acid and charring. (B) m/z values from MALDI-TOF-MS spectra correspond to [M+Na]^+^ adducts of a family of methylated keto-mycolates and α-mycolates for purified lipids X and Y, respectively. (C) For ^1^H-NMR analysis, protons are labelled (a to h) according to their respective positions in functional groups. Relative integrations of protons have been normalized according to the number of ethylenic protons (2 for X and 4 for Y) and are indicated in brackets. *stands for proton ^1^H signals of contaminant ethanol present in the NMR tubes.

We reasoned that a detailed structural analysis of the two products X and Y should help to determine which particular enzymatic step of the mycolic acid biosynthetic pathway is inhibited by these drugs. MAMEs were extracted from with either TAC- or SRI-224-treated cells (5 µg/ml) for 24 h and X and Y were isolated as described in Methods. Purity of the lipids was confirmed by conventional TLC analysis (Figure 4A).

The structures of purified lipids X and Y were determined using mass spectrometry (MS) and NMR spectroscopy. As shown in Figure 4B, lipid X exhibits a MALDI-TOF-MS profile corresponding to [M+Na]^+^ adducts of a family of C_77_ to C_87_ oxygenated mycolates, in agreement with earlier studies [36]. Calculated elemental compositions typify them as either keto-, epoxy- or *ω*-1-methoxymycolates. ^1^H-NMR analysis shows distinctive signals at δ 2.41, 2.50 and 1.05 ppm attributed to -CH2(g) -CH(d) and -CH3(f) associated with a keto-functional group, which establishes the nature of X as a keto-mycolate precursor. However, it also indicates the absence of both *cis*- and *trans*-cyclopropane rings, as the expected characteristic upfield signals between δ -0.5 and 0.7 ppm are missing (Figure 4C). In addition, *cis*-ethylenic protons (a) and methylenic protons (b) adjacent to *cis*-double bonds were observed at δ 5.35 and 2.01 ppm, respectively, indicating that cyclopropane group is replaced by a double bond. Accordingly, relative integrations of specific signals associated with a double bond (a, b), keto group (f, d) and methyl-esterified extremity of mycolate (c, e) indicate the presence of a single keto group and a single double bond per molecule. In contrast to lipid X, which appears to be a keto-mycolate precursor, lipid Y showed a different MS profile, with similarities to a family of C_75_ to C_82 _α-mycolates. As for X, ^1^H-NMR analysis of Y is characterized by the absence of signals assigned to *cis*- and *trans*-cyclopropane rings, and by the presence of intense *cis*-ethylenic protons (a) and methylenic protons (b) adjacent to *cis*-double bonds at δ 5.35 and 2.01 ppm, respectively. Consistent with the MS data, no signals associated with oxygenated functional group (keto, methoxy or epoxy) were observed. Relative integrations of signals clearly established the presence of two double bonds per molecule, thus typifying Y as a di-uncyclopropanated α-mycolate. Structures of TAC/SRI-224-induced lipids X and Y are represented in Figure 1.

### TAC affects mycolic acid cyclopropanation in different mycobacterial strains

The sub-types of mycolic acids and their relative ratios differ with the mycobacterial strain [10]. We examined whether the drug-induced inhibition of cyclopropanation in α- and keto-mycolates with associated accumulation of unsaturated mycolate precursors was species-specific. The responses of two other mycobacterial strains, *M. marinum* and *M. chelonae* that produce different mycolic acid sub-type combinations (Figure 1), to treatment with TAC or SRI-224 were examined.

**Figure 5:**
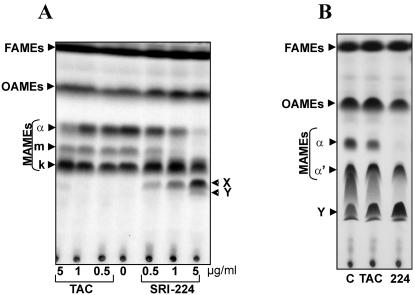
Inhibition of synthesis of cell wall mycolic acids in different mycobacterial species by treatment with TAC or its analogue SRI-224. Autoradiogram of FAMEs and MAMEs extracted from exponentially growing cells that were radiolabeled *in vivo* with ^14^C-acetate. Drug concentrations are indicated in µg/ml. The different mycolates are indicated by arrows. Mycolic acid profiles from *M. marinum* (A) and from *M. chelonae* (B). All other details are as in Figure 3.

*M. marinum*, like *M. tb*, synthesizes three types of mycolates: the dicyclopropanated α-, and the oxygenated keto- and methoxy-mycolates (Figure 1) [37]. Unlike *M. tb*, the oxygenated mycolates lack proximal *trans*-cyclopropanation. Treatment of *M. marinum* cells with SRI-224 appeared to have a more dramatic effect on mycolate synthesis as compared to TAC (Figure 5A). At higher concentrations of SRI-224, the synthesis of the oxygenated mycolates was severely diminished. Treatment with TAC led to slight accumulation of an X-like product, while SRI-224 treatment led to a strong accumulation of X-like and Y-like lipids.

The repertoire of mycolates of *M. chelonae* consists of an unsaturated α' and di-cyclopropanated α-mycolates along with their unsaturated precursors bearing *cis*/*cis* or *cis*/*trans* double bonds [38]. As expected, after treatment with SRI-224 at 20 µg/ml, the synthesis of α-mycolates was largely inhibited and was only accompanied by the accumulation of an unsaturated product, presumably lipid Y (Figure 5B). As observed in the other species, the effect of TAC on α-mycolates was less intense than that of SRI-224 at the same concentration.

Thus, all three mycobacterial species were similarly affected in mycolic acid synthesis in the presence of TAC or SRI-224. The drugs appear to inhibit mycolic acid synthesis, not during the fatty acid elongation which is catalyzed by the type II fatty acid synthase (FAS-II) [9], [10], but rather at the later step of cyclopropanation, thus resulting in accumulation of unsaturated mycolic acids.

### *In vivo* quantification of cyclopropane rings in whole cells

**Figure 6:**
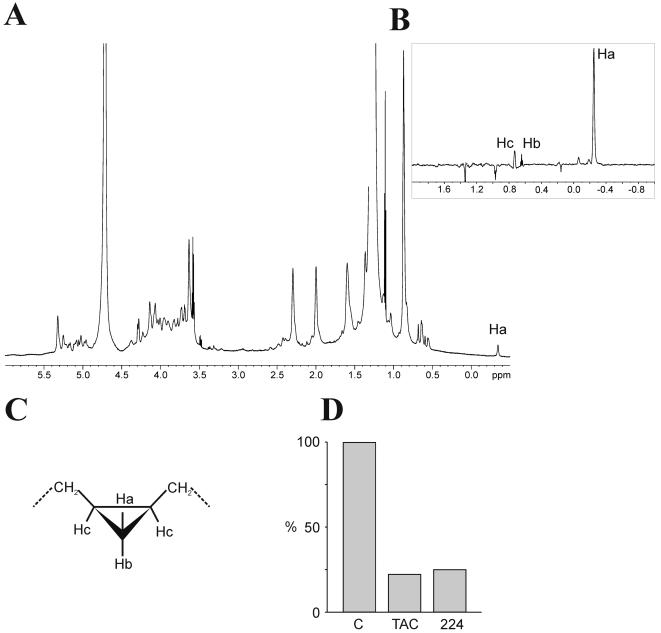
*In vivo* identification and relative quantification of *cis*-cyclopropanes by ^1^H HR-MAS NMR. (A) Detail of ^1^H HR-MAS spectrum of control whole cells of *M. bovis* BCG. (B) Unidimensional selective COSY spectrum after irradiation of Ha signal showing ^3^ *J* and ^2^ *J* connectivities of *cis*-cyclopropyl ring Hb and Hc protons to Ha as depicted in (C). (D) Relative quantification by ^1^H HR-MAS NMR of *cis*-cyclopropanes based on differential integration of the Ha signals in control untreated cells (c) or cells treated with TAC-treated (1 µg/ml) (TAC) or SRI-224-treated (1 µg/ml) (224). Results are representative of two independent experiments.

Relative quantification of mycolate cyclopropanation in whole cells of mycobacteria was assessed by High-Resolution Magic Angle Spinning (HR-MAS) Nuclear Magnetic Resonance (NMR). This technique was previously shown to be effective for directly demonstrating the effects of the antitubercular drug ethambutol on the cell wall polysaccharides of living mycobacteria [39]. Here, for the first time, we apply this method to observe modifications in the structures of mycolic acids directly on whole cells of mycobacteria. The ^1^H HR-MAS NMR spectrum of intact *M. bovis* BCG control cells in D_2_O showed the presence of a well-isolated but excessively broad signal, presumably resulting from the low diffusion rate of mycolates within the cell wall, centred at −0.33 ppm (data not shown). This was tentatively attributed to the upfield methylenic proton of the *cis*-cyclopropyl ring according to standard purified mycolates and literature [40]. As shown in Figure 6A, a well resolved signal (Ha) was obtained after partial disruption of mycobacterial cell wall by adding 5 µl of chloroform to cell pellets directly into the Zr rotor. Attribution of spin system of cyclopropyl ring was confirmed by irradiating Ha proton by a selective COSY experiment. This permitted observation of Hb and Hc protons of the cyclopropyl ring at δ 0.56 and 0.64 ppm through ^3^ *J* and ^2^ *J* connectivities with Ha, respectively (Figure 6B, C). Based on these data, we used Ha proton as a probe for the relative quantification of cyclopropanation by comparing its intensity in control and SRI-224-treated cells of *M. bovis* BCG, after normalizing the spectra on -CH_2_- signal at δ1.25. As shown in Figure 6D, treatment of cultures with 1 µg/ml of TAC or SRI-224 reduced the quantity of *cis*-cyclopropane rings by ∼75%.

Therefore, these data fully support and extend the conclusion that the lipids X and Y are uncyclopropanated counterparts of the oxygenated and α-mycolates, respectively. Interestingly, the structure of these precursors is reminiscent of the mycolate precursors that accumulate in *M. tb* mutant strains carrying a *CMAS* gene deletion, such as in *cmaA2* [41], *pcaA* [12] or *mmaA2* [23].

### Overproduction of CMAS in *M. bovis* BCG partially reverses the effects of TAC

On the basis of the TLC and structural analyses of lipids which accumulate in the presence of TAC or SRI-224, designated here as X and Y, cyclopropanation appeared to be the site of action of the drugs, in particular, cyclopropanation of the proximal double bond. PcaA and CmaA2 have been shown to be responsible for the *cis*- and *trans*-cyclopropanation of the proximal double bond in α- and oxygenated mycolates, respectively [12], [41]. MmaA2 has been reported to catalyze cyclopropanation of the distal double bond in α-mycolates and *cis*-cyclopropanation of the proximal double bond in the oxygenated mycolic acids [23]. If the cyclopropanating activities of these enzymes is being inhibited by TAC, then overproduction of the enzymes should reverse the effects on mycolic acid profile. We tested this possibility by independently overexpressing the *cmaA2*, *mmaA2* or *pcaA* genes of *M. tb* H37Rv both in *M. bovis* BCG and *M. marinum*.

**Figure 7:**
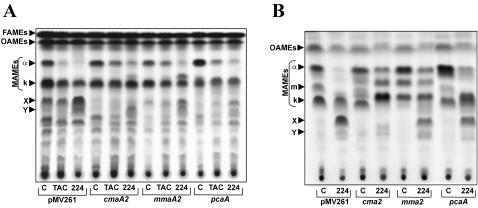
Partial recovery of mycolic acid synthesis in the presence of TAC or SRI-224 in strains overexpressing the *CMAS* genes. Autoradiogram of FAMEs and MAMEs extracted from exponentially growing cells that were radiolabeled *in vivo* with ^14^C-acetate. (A) *M. bovis* BCG or (B) *M. marinum* containing the plasmid vector pMV261 or the same vector carrying *cmaA2, mmaA2* or *pcaA* of *M. tb* H37Rv. Extracts were obtained from untreated control cells (c) or cells treated with 5 µg/ml of either TAC or SRI-224 as indicated. All other details are as in Figure 3.

The mycolic acid profiles of *M. bovis* BCG and *M. marinum* overexpressing the selected *CMAS* genes were analyzed, with or without treatment with 5 µg/ml of either TAC or SRI-224 (Figure 7). The effects were similar in both species. In general, cell overproducing a CMAS enzyme were able to partially resist the drug-induced changes in the mycolic acid profile. The amount of α-mycolates produced under drug treatment in the recombinant strains was detectably higher than in the control strain. More obvious was the diminution of lipids X and Y, especially in the *cmaA2*-overexpressing strain (Figure 7A). In the case of *M. marinum* overexpressing either *cmaA2* or *pcaA*, treatment with SRI-224 led to a decrease in the X-like product and also an increase in the synthesis of keto-mycolates (Figure 7B). In contrast, overexpression of *mmaA2* in this strain had the effect of reducing the amount of drug-induced product X, but there was no detectable increase in keto-mycolates. Thus, while overexpression of no single gene alone permitted full recovery of mycolate synthesis in the presence of the drugs, the accumulation of the lipids X and Y was distinctly reduced. It is very likely, that full recovery of the mycolic acid profile in the presence of the drug would require co-ordinate overexpression of all the relevant *CMAS* genes at appropriate levels.

## Discussion

TAC is a widely used antituberculosis drug that forms a second-line of therapy, often in conjunction with another drug, such as INH [42]. Its use has been controversial due to the occurrence of skin toxicity in HIV-positive individuals [43]. Chemical analogues that may share the antimycobacterial activity of TAC without its toxicity would be attractive in therapy. Based on MIC values of the chemical analogues of TAC against *M. tb* H37Rv, several promising candidate drugs from the series shown in Figure 2 appeared more potent than the parent molecule TAC. Of these, SRI-224 and SRI-286 have earlier been shown to have potent activity against different mycobacterial species both *in vitro* and *in vivo* [5], [6], [44]. Treatment with drugs like INH, ETH, ISO or thiolactomycin inhibits the fatty acid synthase, FAS-II, a biosynthetic cycle contributing the early steps of mycolate synthesis, thus effectively preventing synthesis of all mycolic acid sub-types. TAC does not inhibit FAS-II activity but appears to act at a later step. Once elongation of the fatty acid chain is achieved by FAS-II, the meromycolates fail to be cyclopropanated in the presence of TAC/SRI-224. Nevertheless, this does not prevent them from being condensed with the α-chain *via* the polyketide synthase Pks13 [45] and then covalently linked to sugars like arabinogalactan. A result of TAC/SRI-224 treatment is thus, accumulation of uncyclopropanated mycolic acids in the cell wall.

Whole cell analyses by HR-MAS NMR reveals structural properties of surface-bound molecules. The signal indicating presence of a proximal *cis*-cyclopropane is well-isolated in the NMR spectrum and hence easily identifiable. Taking advantage of this observation, we were able to conclusively demonstrate a decrease in the *cis*-cyclopropane signal in drug-treated mycobacteria. Structural analyses of the unusual lipids, X and Y, which accumulate in drug-treated cells confirmed absence of cyclopropanation in cell wall mycolic acids. Further, as observed by TLC analyses, the mycolate profile could be partially recovered by overexpression of *cmaA2*, *mmaA2* or *pcaA*, singly. Thus, by following complementary experimental approaches, we arrived at the same conclusion that TAC and SRI-224 inhibit CMAS activities. A direct proof employing an *in vitro* assay to monitor the activity of the CMASs in the presence of TAC and its analogues is desirable, but not available to date. This is essentially due to the lack of an experimental ability to synthesize the meromycolyl-ACP substrates, suitably unsaturated at specific sites. Moreover, to demonstrate the effect of TAC on the CMAS activity *in vitro*, it would be mandatory to supply TAC in its activated form, i.e., after the action of the EthA monoxygenase. If a stable, activated form of the drug could be made available *in vitro*, its co-crystallization with a CMAS could also be envisaged.

**Figure 8:**
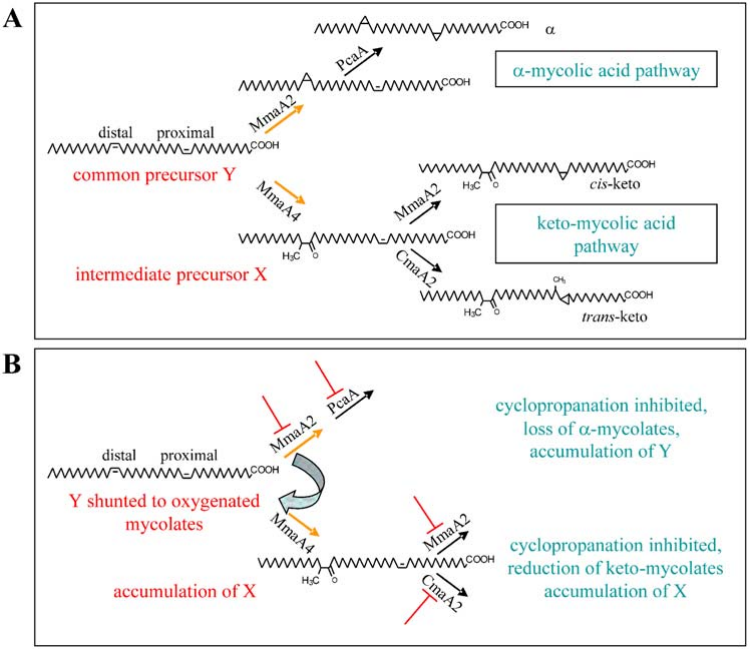
Proposed mechanism for generation of mycolic acid sub-types by the action of CMAS enzymes (A) and inhibition by TAC/SRI-224 (B). The generation of α- and the oxygenated mycolic acids is considered to follow to two independent pathways. A common, di-unsaturated precursor, Y, is envisaged for the two pathways. Y is subsequently transformed into α-mycolic acids by the action of the MmaA2 and PcaA that modify the distal or proximal double bond, respectively. Action of MmaA4 commits Y to the pathway for the oxygenated mycolic acids, by producing the precursor X. MmaA3, which is required for generation of methoxy-mycolic acids in *M. tb* is inactive in *M. bovis* BCG Pasteur due to the presence of a point mutation [50]. The proximal double bond is modified by the CmaA2 (and MmaA2) or PcaA to generate *trans*- or *cis*-cyclopropanated derivatives, respectively. In the presence of TAC, all the CMASs mentioned above are inhibited, except for MmaA4. Due to inhibition of MmaA2, excess of Y is diverted to MmaA4 leading to generation of X, which accumulates due to lack of activities of CmaA2 and MmaA2. SRI-224 appears to affect MmaA4 to a certain degree, leading to accumulation Y in addition to X. (For simplicity, only the meromycolyl moiety of mycolates has been depicted).

Collation of current knowledge of CMAS enzymology and the data presented here on the structures of mycolic acids synthesized in the presence of TAC or its analogues, leads us to propose a model for the action of these drugs. A simplified schematic shown in Figure 8A illustrates generation of the mycolic acid sub-types in *M. bovis* BCG as a result of action of different CMAS enzymes. Lipid Y, which is unsaturated at the distal and proximal positions, can be regarded as a common precursor for generation of both α- and keto-mycolates, and could serve as a substrate for both MmaA2 and MmaA4. MmaA2 would introduce a distal *cis*-cyclopropane and thus commit Y to the α-mycolic acid branch of synthesis, while action of MmaA4 would initiate synthesis of the oxygenated lipid X, committing it to keto-mycolate synthesis [23], [46]. Next, PcaA or CmaA2 would introduce the proximal cyclopropane rings [12], [41], generating either the α- or keto- mycolates, respectively. From the structural analysis of the lipids X and Y, it appears that the CMASs are differentially inhibited by TAC or SRI-224. According to our model (Figure 8B), MmaA2 is most strongly inhibited by TAC, while MmaA4 is largely unaffected. We speculate that in TAC-treated cells, owing to the inhibition of MmaA2 activity, the initial excess of common precursor Y would be shunted to serve as a substrate for MmaA4 resulting in synthesis of X, which accumulates due to lack of activity of CmaA2 and MmaA2. Thus, treatment with TAC led to a more significant reduction in the content of α-mycolates compared to the keto-mycolates with concomitant build-up of X. A similar but opposite situation has been reported in a MmaA4 knock-out mutant [46], wherein the Y is shunted to serve as a substrate for MmaA2 instead, leading to lack of oxygenated mycolates with concomitant accumulation of precursors of α-mycolates. Treatment with SRI-224 appears to inhibit MmaA4 as well as the other CMASs, leading to accumulation of the precursor Y. Thus, despite their high degree of structural homology [21], [47], the CMASs show differential sensitivity to TAC and SRI-224, resulting in abnormal ratios of mycolates.

While our data points overwhelmingly to the inhibition of the CMASs by TAC or SRI-224, overexpression of the CMAS genes as described here was unable to significantly alter the MICs against the drugs (data not shown). Moreover, in other growth experiments, we observed a significant inhibition of cyclopropanation at low concentrations (0.025 µg/ml, ten times lower than the MIC value) without any significant effect on growth in culture (data not shown). This is consistent with the reports that *in vitro* growth of *M. tb* mutants in *cmaA2*, *pcaA*, *mmaA2* or *mmaA4* was largely unaffected, although the virulence of these was compromised in mice and macrophage cell lines [11], [12], [17]. Thus, while the CMASs enzymes show extreme sensitivity to TAC, the lack of cyclopropanated mycolic acids may not be the basis for the growth inhibition *in vitro,* pointing to the existence of additional drug target(s) responsible for the observed bacteriostasis. The success of TAC as an antitubercular drug might, therefore, be attributed to the likely effects on an unknown target(s) as well as to the consequences of inhibition of CMAS activities *in vivo*. It is also possible that the simultaneous inhibition of several members of the CMAS family by TAC as seen in this study, has a more pronounced effect on growth *in vivo* than that caused by the lack of an individual CMAS enzyme through mutation as described by the earlier reports [11], [12], [17]. To resolve the matter, we are currently pursuing a mutagenesis approach to screen for TAC-resistant mutants. The aim is to uncover a strain that would be drug-resistant due to a mutation influencing the alternate target, while still being affected in mycolic acid cyclopropanation like the parent strain. The effect of the drug on mycolates would thus be uncoupled from the effects leading to the bacteriostatic effect *in vitro*. We have isolated a few spontaneous drug-resistant mutants for both TAC and SRI-224, which are currently being analyzed in terms of their mycolate profile, cell wall permeability and immunopathogenicity.

Molar ratios of the mycolic acid sub-types profoundly affect fluidity and permeability of the cell wall, as well as the immunological response in the host [11], [12], [28], [29]. Together, these observations favor the idea of CMAS family being important in virulence and persistence in pathogenic mycobacteria. The absence of cyclopropanated lipids in mammals emphasizes CMASs as attractive drug targets. The presence of persistent bacteria is considered to be the major reason for a lengthy therapy [48]. Therefore, genes such as *pcaA* have been proposed to be attractive target for the development of drugs against persistent bacilli [49]. However, all studies reported so far demonstrate the effects of knock-out of a single CMAS gene at a time, unlike the effect of the drugs described here that are inhibitory to practically the entire family of CMASs, all at the same time. In addition, the structural similarity of these enzymes [21], [47] suggests the possibility that one inhibitor may be effective against multiple targets, reducing the potential for drug resistance, particularly desirable for any new drug in the fight against tuberculosis. In this context, among the second generation TAC analogues described here, those with increased potency may be evaluated for desirable pharmacokinetic properties. Overall, our study presents for the first time a mechanism of action of TAC that could at least partially explain its effectiveness in the field. It likely provides a foundation for rational drug design, which may lead to the development of a novel class of inhibitors targeting the CMAS family of enzymes.
